# Increasing Signal Intensity of Fluorescent Oligo-Labeled Antibodies

**DOI:** 10.1101/2023.07.06.547965

**Published:** 2023-07-06

**Authors:** Madeline E. McCarthy, Danielle R. Latham, Charlotte V. Haskell, Nishi D. Patel, Zachariah A. Pittman, Hugo Sanabria, Marc R. Birtwistle

**Affiliations:** 1 Department of Chemical and Biomolecular Engineering, Clemson University; 2 Department of Physics and Astronomy, Clemson University; 3 Department of Bioengineering, Clemson University

## Abstract

While full-spectrum flow cytometry has increased antibody-based multiplexing, yet further increases remain potentially impactful. We recently proposed how fluorescence Multiplexing using Spectral Imaging and Combinatorics (MuSIC) could do so using tandem dyes and an oligo-based antibody labeling method. In this work, we found that such labeled antibodies had significantly lower signal intensity than conventionally-labeled antibodies in human cell experiments. To improve signal intensity, we tested moving the fluorophores from the original external (ext.) 5’ or 3’ end-labeled orientation to internal (int.) fluorophore modifications. Cell-free spectrophotometer measurements showed a ~6-fold signal intensity increase of the new int. configuration compared to the previous ext. configuration. Time-resolved fluorescence spectroscopy and fluorescence correlation spectroscopy showed that ~3-fold brightness difference is due to static quenching. Spectral flow cytometry experiments using peripheral blood mononuclear cells stained with anti-CD8 antibodies showed that int. MuSIC probe-labeled antibodies have signal intensity equal to or greater than conventionally-labeled antibodies with similar estimated proportion of CD8+ lymphocytes. The antibody labeling approach is general and can be broadly applied to many biological and diagnostic applications.

## Introduction

Fluorescent antibodies are an integral tool for biological and diagnostic applications^1^. One application for fluorescent antibodies is flow cytometry^2^. The use of fluorescent antibodies with conventional flow cytometers is restricted to typically 3–4 markers, but up to ~10–15 markers have been reported^2–4^. The restriction is largely due to spectral overlap between fluorophores, limiting the number of analytes that can be reliably detected. Regardless, flow cytometry remains a useful platform as it is a cost-effective, high-throughput, and non-destructive method for single-cell analysis^5,6^. Recent advances have led to full-spectrum flow cytometry (FSFC), which captures the entire fluorophore emission spectra, creating a unique spectral fingerprint for each fluorophore^7,8^. This allows fluorophores with similar peak emissions to be used in the same panel, so long as they have distinctive spectral signatures. FSFC has enabled the detection of up to 40 markers simultaneously9, but further multiplexing capabilities are stunted by the number of commercially available dyes that are compatible in a single panel. Moreover, FSFC is still far from the multiplexing capabilities of methods such as single-cell RNA sequencing, which has the ability to identify 100s-10,000s of markers^10,11^.

**Figure 1.**
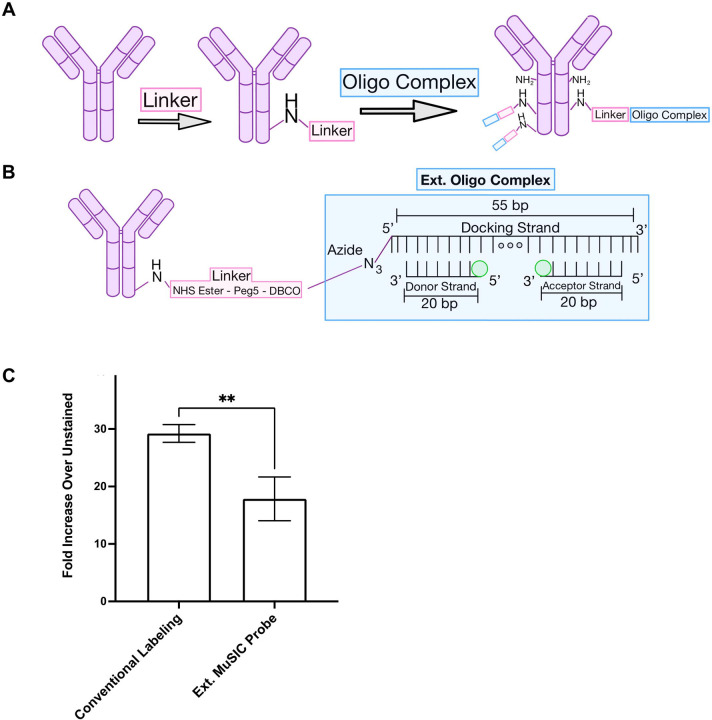
Oligo-based MuSIC probe labeling of antibodies. (A-B) Graphic depicting MuSIC probe labeling. By reacting the NHS ester of the linker with free amines on the antibody, the linker is attached. Fluorophore-labeled donor and acceptor strands are annealed onto the docking strand to form the oligo complex. The azide on the docking strand, in the oligo complex, is reacted with the free DBCO group on the linker to covalently bind the oligo complex to the antibody. There are multiple free amines on each antibody, allowing for the linker to attach at multiple sites, increasing the degree of labeling. (C) Comparison of fluorescence intensity of PBMCs stained with CF488A conventional labeling kit vs Atto488 Ext. MuSIC probes. The fold increase in intensity over unstained cells is significantly greater for the conventional labeling kit compared to the ext. MuSIC probe. Error bars are standard error from triplicate measurements.

The 40-plex FSFC panel largely relies on single-dye fluorescent antibodies, with relatively few tandem-dye fluorescent antibodies^9^. We recently developed Multiplexing using Spectral Imaging and Combinatorics (MuSIC), which uses combinations of currently available fluorophores to create spectrally-unique MuSIC probes^12^. MuSIC probe-labeled antibodies may expand the multiplexing capability for FSFC by providing new tandem probes. Previously, we proposed an oligo-based method for covalently labeling antibodies with MuSIC probes (Fig 1A–B) and validated this method using spin column purification, absorbance measurements, and Protein A beads / spectral flow cytometry13. However, this method had yet to be tested on human cells.

In the current study, we first applied our previous method to staining human peripheral blood mononuclear cells (PBMC). In doing so, we found significantly lower staining intensity compared to a conventional antibody labeling kit (Biotium Mix-n-Stain). Consequently, we hypothesized that a different oligo-fluorophore arrangement of the MuSIC probes, using internal fluorophore modifications rather than external fluorophore modifications, could increase the fluorescent signal intensity of MuSIC-probe labeled antibodies. Results showed that the new method with internal fluorophore modifications produced ~6-fold increase in fluorescent signal compared to the previous method. Biophysical characterization showed that ~3-fold of this difference is due to fluorescence static quenching. We then compared the internally modified MuSIC-probe labeled antibodies to conventionally labeled antibodies by staining PBMCs. Results showed that the new internal labeling method has ~2-fold increase in fluorescent signal over the conventionally labeled antibodies while having no significant difference in the estimated % of CD8+ lymphocytes. This increased fluorescent signal suggests the potential of MuSIC-probe labeled antibodies to add to the existing capabilities of FCFS, by providing new spectrally unique fluorescent antibodies with comparable intensity. Such antibodies are not restricted to FSFC but could be useful for other biomedical applications such as tissue heterogeneity studies with immunofluorescence imaging when spectral detection is available.

## Methods

### Measuring fluorescent oligo emission spectra

**Figure 2.**
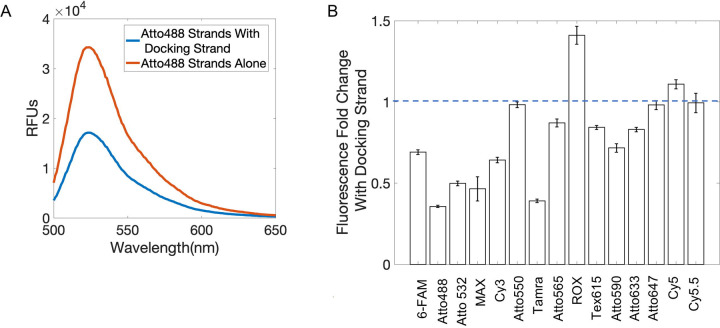
Fluorescence signal change from Docking Strand. (A) Comparison of fluorescence emission spectra, excited at 470nm, of the Atto488 5’ Donor and 3’ Acceptor strands hybridized to the Docking Strand and when alone in solution with and without the Docking Strand. Data are representative from triplicates. (B) Change in fluorescence intensity of 15 fluorescent oligos when hybridized to the Docking Strand. Error bars are standard error from triplicates.

All oligos (Integrated DNA Technologies, Table 1) are resuspended in ddH20 at 100 μM. In a black 96-well plate (Fisher Scientific Cat: 655900), 200 μmols of the fluorescent oligo(s) is added to the well and the volume is brought up to 50μl with PBS. The fluorescent emission spectra are gathered using a Synergy MX microplate reader (Biotek) with parameters set to a slit width of 9 nm, taking readings from the top, an excitation wavelength set to the maximum excitation wavelength for that fluorophore, and an emission wavelength starting 30 nm after the excitation wavelength (Table 2) and emission collected at every nm. The maximum emission intensity was used to quantify results in Fig. 2B.

### Labeling Antibodies

Antibodies are conjugated as previously described^13^. In short, the antibody (CD8 clone RPA-T8; Biolegend Cat: 301002) is incubated with DBCO-Peg5-NHS Ester (linker; 10mM in DMSO; Click Chemistry Tools Cat: 1378531-80-6) in 60 molar excess (10 μg of antibody and 2.8 μg of linker) for 30 minutes at room temperature. Post-incubation, the excess linker is removed with Amicon Ultra 100 kDa molecular weight cut-off filters (Fisher Scientific Cat: UFC5100BK). The antibody-linker retentate is collected. Two oligo complexes are created using external (ext.) or internal (int.) fluorophore modifications.

For externally-modified oligos, a 20 bp oligo with a 5’ fluorophore modification (5’ donor strand) and a 20 bp oligo with a 3’ fluorophore modification (3’ acceptor strand) are co-hybridized to a 55 bp oligo with a 5’ azide modification (docking strand) (Integrated DNA Technologies, Table 1) in a 1:1:1 ratio (0.4 nmol of each oligo) to form the ext. oligo complex.

For internally-modified oligos, a 15bp oligo with a 3’ azide modification (azide strand) and a 50 bp oligo with an internal fluorophore modification (int. acceptor strand) are co-hybridized to a 65 bp oligo with an internal fluorophore modification (int. donor strand) (Integrated DNA Technologies, Table 2) at a 1:1:1 ratio to one another (0.4 nmol of each oligo) to form the int. oligo complex.

For each, oligo mixtures are incubated for five minutes at room temperature in the dark to allow for complex formation. These complexes (0.4 nmol of each oligo) are then added to the antibody-linker retentate at a 6-molar excess to the original 10 ug of antibody. The volume is brought up to 100 μl with PBS and incubated at 4°C overnight in the dark.

Conventionally labeled antibodies are labeled as per the manufacturer’s instructions (Biotium, Cat: 92446). In short, CD8 antibodies are covalently labeled with CF488A dyes using the Biotium mix-n-stain kit.

### Preparing Peripheral Blood Mononuclear Cells

Normal Peripheral Blood Mononuclear Cells (PBMCs) (Precision for Medicine; 10M cells/vial) are thawed and counted with a hemacytometer. Cells are washed twice with cold (4°C) stain buffer (0.01 g/ml BSA in PBS) at 300 x g for 5 min. Post-wash, the cells are resuspended in cold stain buffer and divided into 100 μl aliquots containing 10^6^ cells.

### Staining PBMCs

In order to block non-specific Fc-mediated interaction,1 μg of normal Rabbit IgG (ThermoFisher Cat: 31235) is added to the cell sample and incubated for 10 minutes at room temperature. Conventionally, ext., and int. labeled-antibodies are made for staining using the protocols described above (10μg of antibody each); (1) CD8 (clone RPA-T8; Biolegend Cat: 301002) labeled with Atto488 ext. MuSIC probes, (2) CD8 (clone RPA-T8; Biolegend Cat: 301002) labeled with Atto488 int. MuSIC probes, and (3) CD8 (clone RPA-T8; Biolegend Cat: 301002) labeled with CF488A (Biotium Cat: 92446), Antibody concentration is adjusted to 0.25 ug/ul for each sample. The labeled CD8 antibody is added to the cell sample at the appropriate amount as per manufacturer’s recommendations (2μg CD8 antibody / 10^6^ cells) and allowed to incubate in the dark for 20 minutes on ice. Post-incubation, cells are washed twice with 1 ml of cold staining buffer at 300 x g for 5 min. The final cell pellet is resuspended in 0.5 ml of cold staining buffer.

### Flow Cytometry

Stained PBMC samples are analyzed using a Cytek Aurora spectral flow cytometer. First, unstained PBMCs are assayed with the events to record set to 10,000. The SpectroFlo software (Cytek) is used to gate single cells (lymphocytes, monocytes, and granulocytes) by forward and side scatter. We then further gate specifically over the lymphocyte population, as typical based on light scattering distributions^14^. Using these same settings, the stained cell samples are assayed. To compare fluorescence intensity between stained samples we calculate the median intensity of the positively stained cells in the maximum emission channel (B2) using the Spectroflo software. Positively stained cells are defined as cells with a staining intensity above that of the unstained cell samples using a marker gate.

### Size Exclusion Chromatography / Multi-Angle Light Scattering (SEC-MALS)

The purpose of SEC was to purify labeled antibody samples prepared as above to provide monomeric antibody conjugates for biophysical characterization below. The approximate retention behavior and molar mass determination of the SEC column and MALS detector (Agilent, AdvanceBio PL1180-3301) was estimated first using Bovine Serum Albumin at 0.5 mg/mL, 30 injection, and a 0.30 mL/min flowrate using a PBS mobile phase. The MALS instrument (Wyatt Technologies, DAWN 785nm) was normalized, aligned, and broadened using the main peak of the eluent BSA, corresponding to unaggregated BSA (~5 min retention time). The online concentration was determined using a refractive index detector (Wyatt Technologies, Optilab WREX-08), and we assigned each sample a *dn/dc* of 0.185. We injected the labeled antibody solutions prepared as described above using the same conditions as the BSA experiment. As the approximate absolute molar mass determination via MALS indicated (Fig. S1), the chromatogram showed two distinct regions. Eluent corresponding to the first region between 2.5 and 3.5 minutes elution time had an approximate molar mass range of that expected for antibody-oligo conjugates (with a degree of labeling spectrum approximately between 1 and 6). This fraction was collected into vials and preserved for further analysis.

### Fluorescent Correlation Spectroscopy and Time-Resolved Fluorescence and Anisotropy

Freely diffusing samples diluted to sub-nM concentration were analyzed using a custom-built confocal microscope^15^. Samples were excited with a 485 nm pulsed diode laser (LDH-D-C-485, PicoQuant, Germany) operated at 40 MHz. The laser power at the objective was 141 µW. Emission was detected via two PMA detectors (PicoQuant, Germany), allowing for separation into parallel and perpendicular polarization components. A clean-up emission filter (ET525/50, Semrock) is placed before each detector. To ensure temporal data registration of the two synchronized emission channels, we used a HydraHarp 400 TCSPC module (PicoQuant, Germany) in Time-Tagged Time-Resolved mode with a resolution of 1 ps.

Samples were imaged in NUNC chambers (Lab-Tek, Thermo Scientific) that were pre-coated with a solution of 0.01% Tween 20 (Thermo Scientific) in water for 30 min to minimize surface adsorption. Before measurements, chambers were rinsed with buffer to ensure clean measurements. The instrument response function (IRF) was found by measuring water while the protein-free buffer was used for background subtraction. Samples were diluted in charcoal-filtered PBS (10 mM sodium phosphate, pH 7.4, 137 mM NaCl, 2.7 mM KCl) to ~500pM and measured for 2 minutes.

Software correlations were fit with a 3-dimensional Gaussian with two triplet terms (Eq. 1). The confocal geometric parameter ($\omega_{o}$) was determined using Rhodamine 110 as a standard. Diffusion time ($t_{diff}$), molecule count (N), baseline term ($G_{\infty }$), dark state times ($t_{T_{1}}$ and $t_{T_{2}}$), and their corresponding fractions ($A_{T_{1}}$ and $A_{T_{2}}$) were considered as free parameters.


(1) $$G\left(t_{c}\right)=G_{\infty }+\frac{1}{N}\cdot \frac{1}{1+\frac{t_{c}}{\left|t_{diff}\right|}}\cdot \frac{1}{\sqrt{\left(1+\frac{t_{c}}{\omega_{o}\left|t_{diff}\right|}\right)}}\cdot \left(1-\left|A_{T_{1}}\right|+\left|A_{T_{1}}\right|e^{-\left(\frac{t_{c}}{\left|t_{T_{1}}\right|}\right)}-\left|A_{T_{2}}\right|+\left|A_{T_{2}}\right|e^{-\left(\frac{t_{c}}{\left|t_{T_{2}}\right|}\right)}\right)$$

Fluorescence lifetime and rotational correlation times for the samples were found by determining the minimum number of free parameters required to minimize the χ^2^. This was determined to be two fluorescence lifetimes and one rotational time for each sample. The comparison between parallel and perpendicular polarized light about the original laser pulse gives the time-resolved fluorescence anisotropy (r(t) – Eq. 2). The time-resolved anisotropy was fit to Eq. 3.


(2) $$r\left(t\right)=\frac{I_{parallel\left(t\right)-\left(\frac{1}{G}\right)}I_{perpendicular\left(t\right)}}{I_{parallel\left(t\right)+\left(\frac{2}{G}\right)}I_{perpendicular\left(t\right)}}$$

(3) $$r\left(t\right)=r_{\infty }+\left(r_{o}-r_{\infty }\right)e^{\left(-\frac{t}{\tau_{r}}\right)}≅r_{o}e^{\left(-\frac{t}{\tau_{r}}\right)}$$

For lifetime fitting, the parallel and perpendicular components were combined using the G-factor (G=1.04) determined using Rhodamine 110 as a standard. Then the fluorescence decays were fit using Eq. 4 with two fluorescence lifetimes for minimizing χ^2^. Then Eq. 5 was used to determine the species average lifetime.


(4) $$I\left(t\right)=\Sigma \;A_{n}e^{-\left(\frac{t}{\tau_{n}}\right)}$$

(5) $${\langle \tau \rangle }_{x}=\Sigma \;A_{n}\cdot \tau_{n}$$

## Results

We previously developed a method for labeling antibodies with combinations of fluorophores (i.e. MuSIC probes)^13^. In short, an oligo complex containing fluorescent molecules is conjugated to the antibody via a DBCO-Peg5-NHS ester (referred to as the linker) (Fig 1A). Here the oligo complex is composed of a 20 bp oligo with a 5’ fluorophore modification (referred to as the 5’ donor strand) and a 20 bp oligo with a 3’ fluorophore modification (referred to as the 3’ acceptor strand) that are co-hybridized to a 55bp oligo with a 5’ azide modification (referred to as the docking strand) to form the externally labeled (ext.) oligo complex (Fig 1B). We previously demonstrated our ability to covalently label antibodies with MuSIC probes using this method and validated the labeling protocol with spin-column purification, absorbance measurements, and FSFC measurements with protein A beads bound to (i) Cy3, (ii) Tex615, and (iii) Cy3-Tex615 ext. oligo labeled antibodies^13^.

Since this method had only been validated using beads, we asked whether this method would work when staining peripheral blood mononuclear cells (PBMCs)? We created an ext. oligo complex using an Atto488 5’ donor strand and an Atto488 3’ acceptor strand as the MuSIC probe and conjugated it to anti-CD8 antibodies. For comparison, we used a commercially available Biotium Mix-n-Stain kit to conventionally label CD8 antibodies with CF488A dye, which is reported to have comparable fluorescent properties (excitation peak, emission peak, and brightness) to Atto488^16^. PBMCs were stained with each antibody batch and analyzed by FSFC. Results showed that the median signal intensity of cells stained with the ext. labeled MuSIC probe was ~2-fold (p-value=0.01) lower compared to cells stained with conventionally labeled antibodies (Fig 1C).

We then asked how we can increase the signal intensity of MuSIC probe-labeled antibodies. We reasoned that the lower fluorescence signal was not due to the degree of labeling because it was previously calculated to be within the standard range^13,17^. Some degree of difference in signal intensity may be due to differences in dye properties between CF488A and Atto488, although as mentioned above, the dyes have similar characteristics. To investigate whether the docking strand and/or hybridization played a role, we examined the fluorescence emission intensity of Atto488 5’ donor strands and Atto488 3’ acceptor strands alone in solution and when co-hybridized to the docking strand (Fig 2A). We found that the hybridization of the 5’ donor and 3’ acceptor strands to the docking strand results in a significant decrease in fluorescent signal, as compared to the strands on their own.

We further wondered whether this was a fluorophore-specific phenomenon or if it occurred for other fluorophores. Therefore, we compared the emission intensity with and without docking strand for 15 different fluorophore-conjugated 5’ donor strands and 3’ acceptor strands (Fig 2B). Signal decreased with docking strand for all but five of the fluorophore-conjugated strands that were tested. Previous studies showed that there can be a significant change in fluorescence when oligo-strands containing an end-fluorophore modification are hybridized to strands containing an overhang^18^, such as in our ext. oligo complex.

**Figure 3.**
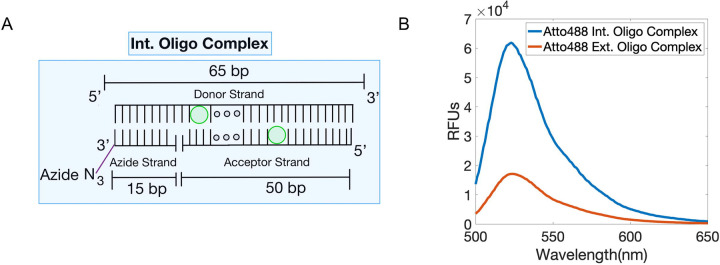
The int. labeling method increases intensity relative to the ext. labeling method. (A) Int. oligo complex containing the Int. Donor and Acceptor strands and the Azide strand. (B) Comparison of relative fluorescence intensity of the Atto488 probes using the int. and ext. oligo complexes (470 nm excitation). Data are representative from triplicates.

These findings led us to hypothesize that if the fluorophores within the oligo complex had a different orientation, it could give an increased fluorescent signal. To test this, we adjusted the configuration of the ext. oligo complex to contain oligos with internally (int) conjugated fluorophores. This new oligo complex consists of the 50 bp int. acceptor strand and a 15 bp azide strand which both co-hybridize to the 65 bp int. donor strand (Fig 3A). The purpose of a separate azide strand is to reduce the cost of oligo production, due to the increased difficulty of synthesizing an oligo with two modifications. The new donor and acceptor strands both have an internal fluorophore modification (int donor and int acceptor), rather than 5’ and 3’ end fluorophore modification, respectively. We then created int. and ext. oligo complexes (both using Atto488 conjugated strands) and measured their fluorescent emission spectra. We observed a ~6-fold fluorescent signal increase of the int. oligo complex compared to the ext. oligo complex in solution (Fig 3B).

**Figure 4.**
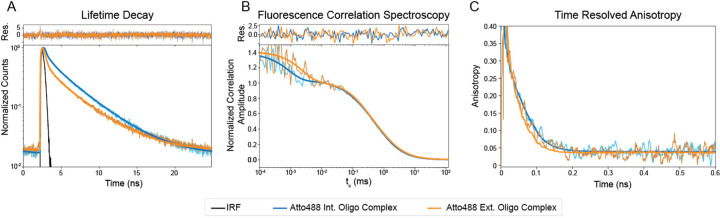
Differentiating static and dynamic quenching by Time-Resolved Fluorescence and Fluorescence Correlation Spectroscopy. Atto488 int. oligo complex fitting is shown in blue, with the raw data in light blue and Atto488 ext. oligo complex fitting is shown in orange, with the raw data in light orange. (A) Normalized fluorescence decays between Atto488 in oligo complex and Atto488 ext. oligo complex. The difference in fluorescence lifetimes is visible by the difference in the slope of the decays. Residuals for the fitting model are shown on top. (B) Normalized fluorescence correlation between Atto488 in oligo complex and Atto488 ext. oligo complex. The difference in dark triplet states is visible in the offset between the start of the curves to correlation times (t_c_) ~10^−3^ ms. The curve overlaps between t_o_ being 10^−1^ and 10, indicating similar diffusion coefficients between samples. Residuals for the fitting model are shown on top. (C) Anisotropy rotational correlation between Atto488 int. oligo complex and Atto488 ext. oligo complex. The slight offset in rotational times could be due to differences in flexibility of the int. and ext. oligo complexes.

To understand the fluorescent signal differences between the int. oligo complex and ext. oligo complex, we used Time Resolve Fluorescence Spectroscopy (TRFs) and Fluorescence Correlation Spectroscopy (FCS) (Fig 4). We found that the ext. oligo complex undergoes more dynamic quenching than the int. oligo complex, as shown by the differences in the fluorescence decay, which can also be quantified using the species average lifetime (Fig 4A, Table 3). The ext. oligo complex also spends more time in the dark triplet state than the int. oligo complex, as shown by the differences in the correlation curves (Fig 4B, Table 3). Further, since FCS only monitors bright molecules, a single (bright) int. oligo complex is only 1.6 times brighter than an ext. oligo complex (Table 3). Considering the observed ~6-fold difference in intensity (Fig. 3B) but < 2-fold difference in the molecular brightness (Table 3) of the int. oligo complex relative to the ext. oligo complex, we conclude that ~3-fold difference in intensity is due to static quenching in the ext. oligo complex. As expected, both oligo complexes show similar diffusion and rotational correlation times (Fig 4B and C).

**Figure 5.**
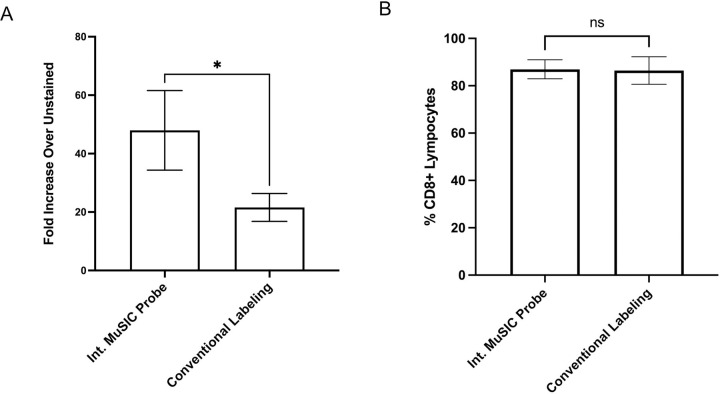
Comparing in oligo complex and conventionally labeled antibodies in cell-based experiments. (A) Fold median signal increase of PBMCs stained with Atto488 int.-labeled CD8 antibodies or CF488A conventionally-labeled CD8 antibodies over unstained PBMCs. (B) Percentage of CD8+ lymphocytes in PBMC for int.-labeled CD8 antibodies compared to CF488A conventional-labeled CD8 antibodies. Measurements are in triplicate and error bars are standard error.

With this increase in signal intensity, we then asked how new int. MuSIC probe-labeled antibodies would compare to conventionally labeled antibodies when staining PBMCs for estimation of specific cell type abundances. Similar to above, int. oligo complexes with Atto488 were conjugated to CD8 antibodies to create int. MuSIC probe-labeled antibodies and CF488A was conjugated to CD8 antibodies using a Mix-n-stain kit to create the conventionally labeled antibodies. PBMCs were stained with each antibody batch and analyzed by FSFC. The signal intensity of cells stained with the int. labeled MuSIC probe was ~2 fold (p-value=0.03) higher compared to cells stained with conventionally labeled antibodies (Fig 5A). When comparing the % of CD8+ lymphocytes detected, we found no significant difference between the int. MuSIC probe-labeled antibodies and conventionally labeled antibodies (Fig 5B). These results demonstrate that we were able to improve the design of MuSIC-probe labeled antibodies to increase the signal-to-noise ratio, with staining behavior comparable to conventionally labeled antibodies.

## Discussion

Here, we established a method to conjugate two fluorophores to an antibody and stain human cells with an increased signal intensity compared to our previous method and conventionally labeled antibodies, with accurate detection of % of CD8+ lymphocytes. This method builds on our previously established labeling protocol but introduces key modifications to the oligo-fluorophore arrangement of the MuSIC probe. By re-arranging the oligo complex to eliminate the use of 3’ or 5’ end fluorophore modifications, we observe a significant increase in fluorescent signal for Atto488. Given the prevalence of docking strand-induced signal decrease, we expect this result may often apply to other fluorophores. We used Time Resolve Fluorescence Spectroscopy and Fluorescence Correlation Spectroscopy to compare the old ext. oligo complex to the new int. oligo complex, finding that increased dynamic quenching and time in the dark triplet state explains the decreased fluorescence intensity of the ext. oligo complex. Using the new design, we stained human PBMCs and compared the signal intensity to that of conventionally labeled fluorescent antibodies, and observed a statistically significant increase in the resulting fluorescent signal without creating any significant differences in the % of CD8+ lymphocytes.

To maximize the potential of this new increased intensity probe design, the next step will be to select different combinations of fluorophores to assemble a palette of spectrally unique antibody-conjugated MuSIC probes. Approaches to do so can include stimulation studies for compatibility using a workflow similar to that described in our previous work^19^, and then testing the highest-ranked fluorophore combinations experimentally. For these simulations, the emission spectra of each possible MuSIC probe is generated, and lists of MuSIC probes that are likely to be deconvolvable in a mixture are generated and ranked. This ranking provides prioritization for testing experimentally by measuring the emission spectra of mixtures of MuSIC probes and unmixing them to determine which MuSIC probes can be accurately demultiplexed.

One major application of using MuSIC probe-labeled antibodies with FSFC can be cell-type profiling, which is the process by which a complex mixture of cell types, for example, from blood or tumors, are classified into the fractional composition of its components (e.g., neutrophils, natural killer cells, various types of T and B cells, etc.), based on classification of expression patterns (e.g., CD3 expressed or not)^20^. While there are 40 FSFC dyes available, very few of them are tandem dyes that can be used as uniquely identifiable markers, which limits the number of individual analytes that can be classified simultaneously. However, MuSIC probe-labeled antibodies could be used to expand the number of markers that can be detected by creating new combination fluorophore probes from the current dyes, to enhance current cell-type profiling efforts. FSFC has been previously paired with cell-type profiling to investigate the correlation between CD38 expression in macrophages and the predicted immune response to immune-checkpoint blockade therapy for hepatocellular carcinoma^21^. With a larger palette of compatible fluorescent tags, cell-type profiling efforts could expand further to look at an increased number of cell-type markers, for a more comprehensive view of a patient’s immune response to various treatments, or to complement other single cell profiling efforts^22–24^.

Additionally, MuSIC probe-labeled antibodies can be applied to a broad range of biological and diagnostic applications that involve the detection of protein expression. One of these applications can be for tissue imaging, such as recent highly multiplexed efforts^25–30^. If MuSIC probe-labeled antibodies can be combined with spectral imaging, this could allow for highly multiplexed, quantitative tissue imaging. One potential application is cancer, where increasing multiplexing capabilities could improve diagnostic potential by allowing for more tumor markers to be analyzed, thus leading to an increased mapping of tumor heterogeneity^31^. This could impact tumor detection, diagnosis, and treatment.

Although here we focused on increasing the fluorescent signal of oligo-based probes, by titrating the fluorescent oligos, we can decrease the fluorescent signal to a desired level in a controllable manner. Tunable fluorescence intensity is useful; for example, in static light scattering experiments^32^, where the sensitive photodiode detectors are easily saturated. Here, they labeled BSA at varying concentrations of fluorescent oligos between 0.03 – 0.10 µM that fluoresced below the saturation limit of the detectors while still achieving desired intensity. Conventional labeling kits would have been too intense, and as most are single reaction use, they can be difficult to control compared to the reported oligo-based probes which offer the unique advantage of reduced, tailorable intensities. In their case, the intensity tunability of the probes enabled a more flexible experimental design capable of separating simultaneous fluorescence and light scattering signals. The tunability of oligo-based fluorescent probe intensity could also be beneficial for cell staining, where some epitopes may have such a high abundance that a reduced fluorescent signal is necessary.

In addition to tunability for probe fluorescence intensity, the new int. oligo arrangement of these probes offers modulation of Förster Resonance Energy Transfer (FRET) between fluorophore combinations on the donor and acceptor strands. By adjusting the distance (bp) between the two fluorophores, one can increase or decrease the FRET efficiency. By adjusting the FRET efficiency of each combination, there is the potential to increase the number of possible probes even further by creating linearly independent combinations.

We conclude that by using an oligo-based approach with internally-labeled fluorophores, we can increase the signal intensity of MuSIC-probe labeled antibodies. MuSIC probe-labeled antibodies may prove useful to increase multiplexing capabilities of full spectrum flow cytometry, and also more broadly where increased multiplexing at single-cell or sub-cellular resolution is needed, including cell-type profiling, tissue studies, and immunofluorescence imaging.

## Supplementary Material

Supplement 1

## Figures and Tables

**Table 1:** Sequences for the ext. and int. oligo complexes.

	Component	Sequence
**Ext. Oligo Complex**	Docking Strand	5′-azide-GTG TAG TTC AGG TCA AGA CAT CGT GCG ACC AGT CAG CAT GAG ACT CAT TGG TGCG-3′
	5’ Donor Strand	3′-C AAG TCC AGT TCT GTA GCA C-fluorophore-5′
	3’ Acceptor Strand	3′-fluorophore-CA GTC GTA CTC TGA GTA AC-5′
**Int. Oligo Complex**	Azide Strand	3’-azide-CGT TAT GAA CCT GA 5’
	Int. Donor Strand	5’ GCA ATA CTT GGA CTA GTC TAG GCG AAC GTT TAA GGC GAT TCT TGT T-fluorophore-A CAA CTC CGA AAT AGG CCG 3’
	Int. Acceptor Strand	3’ CAG ATC CGC TTG CAA ATT CCG C-fluorophore-A GAG ACA AAT GTT GAG GCT TTA TCC GGC 5’

**Table 2:** Fluorophore modifications for donor and acceptor strands with their corresponding excitation wavelength and emission start wavelength.

Fluorophore Modification	Excitation (nm)	Emission (nm)
6-FAM (Fluorescein)	490	520
Atto 488	492	522
Atto 532	524	554
MAX (NHS Ester)	527	557
Cy3	534	564
Atto 550	545	575
Tamra (NHS Ester)	553	583
Atto 565	561	591
ROX (NHS Ester)	578	608
TEX 615	583	613
Atto 590	594	624
Atto 633	623	653
Atto 647	632	662
Cy5	638	668
Cy5.5	676	706

**Table 3:** Fluorescence Correlation Spectroscopy, Time-Resolved Fluorescence and Anisotropy Parameters

	Atto488 Ext. Oligo Complex	Atto488 Int. Oligo Complex
Overall Results		
Molecular Brightness (kHz/molecule)	8.1	13.5
Species Average Lifetime (${\langle \tau \rangle }_{x}$) (ns)	2.58	3.05
Diffusion coefficient (μm^2^/s)	100	120
Quantum Yield	50%	60%
		
Time Resolved Fluorescence and Anisotropy Parameters		
A_1_	57%	68%
t_1_ (ns)	4.0	4.0
A_2_	43%	32%
t _2_ (ns)	0.76	0.92
χ^2^	1.65	1.64
Rotational Time (ps)	280	350

Fluorescence Correlation Spectroscopy		
G_0_	1.00	1.00
N	0.49	0.50
t_diff_ (ms)	0.71	0.59
ω_o_	4.73	4.73
$A_{T_{1}}$	0.28	0.26
$t_{T_{1}}\left(\mu \text{s}\right)$	2.4	0.9
$A_{T_{2}}$	0.13	0.082
$t_{T_{2}}\left(\text{ms}\right)$	0.19	0.088
χ^2^	0.96	0.86
