# Supplementary material for: Increasing Signal Intensity of Fluorescent Oligo-Labeled Antibodies to Enable Combination Multiplexing
Source: bioRxiv. 2024 May 30:2023.07.06.547965. Originally published 2023 Jul 6. Preprint. [Version 2] doi: 10.1101/2023.07.06.547965 (PMC10350089; doi:10.1101/2023.07.06.547965)
Supplement: Supplement 1 [file NIHPP2023.07.06.547965v2-supplement-1.pdf]

bioRxiv preprint doi: https://doi.org/10.1101/2023.07.06.547965; this version posted May 30, 2024. The copyright holder for this preprint (which was not certified by peer review) is the author/funder, who has granted bioRxiv a license to display the preprint in perpetuity. It is made available under aCC-BY 4.0 International license.

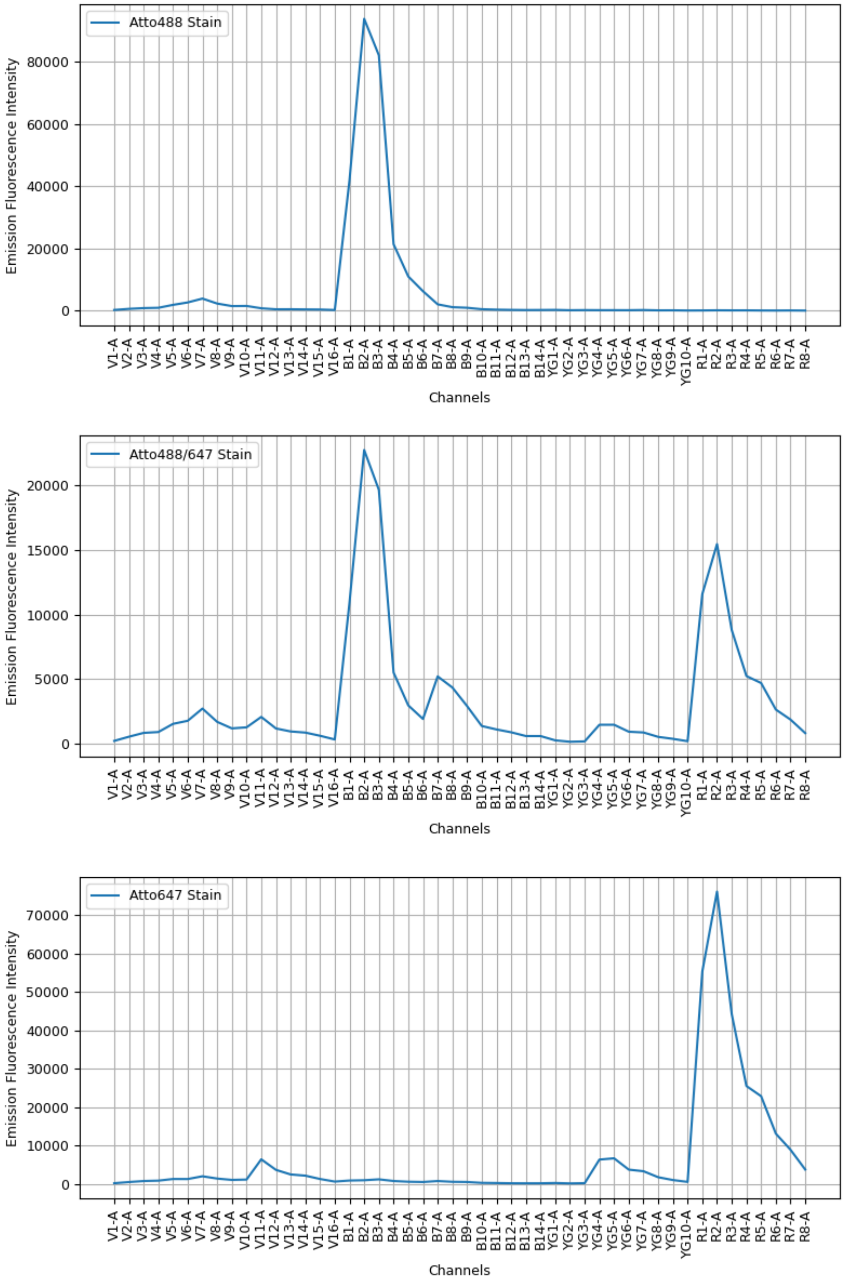

Figure S1. Emission Spectra of the Different Probes. Fluorescence intensities of cells in both unstained and stained-cell groups were measured, and raw data were exported. The median fluorescence intensity (MFI) of each channel in the unstained cells was considered as the autofluorescence. By subtracting this autofluorescence from the corresponding channel's fluorescence value in the stained cells, the true signal of each stained cell was obtained. The MFI of this true signal of each channel was utilized for spectrum plotting of the stained-cell group.

bioRxiv preprint doi: https://doi.org/10.1101/2023.07.06.547965; this version posted May 30, 2024. The copyright holder for this preprint (which was not certified by peer review) is the author/funder, who has granted bioRxiv a license to display the preprint in perpetuity. It is made available under aCC-BY 4.0 International license.

McCarthy et al., Figure S2

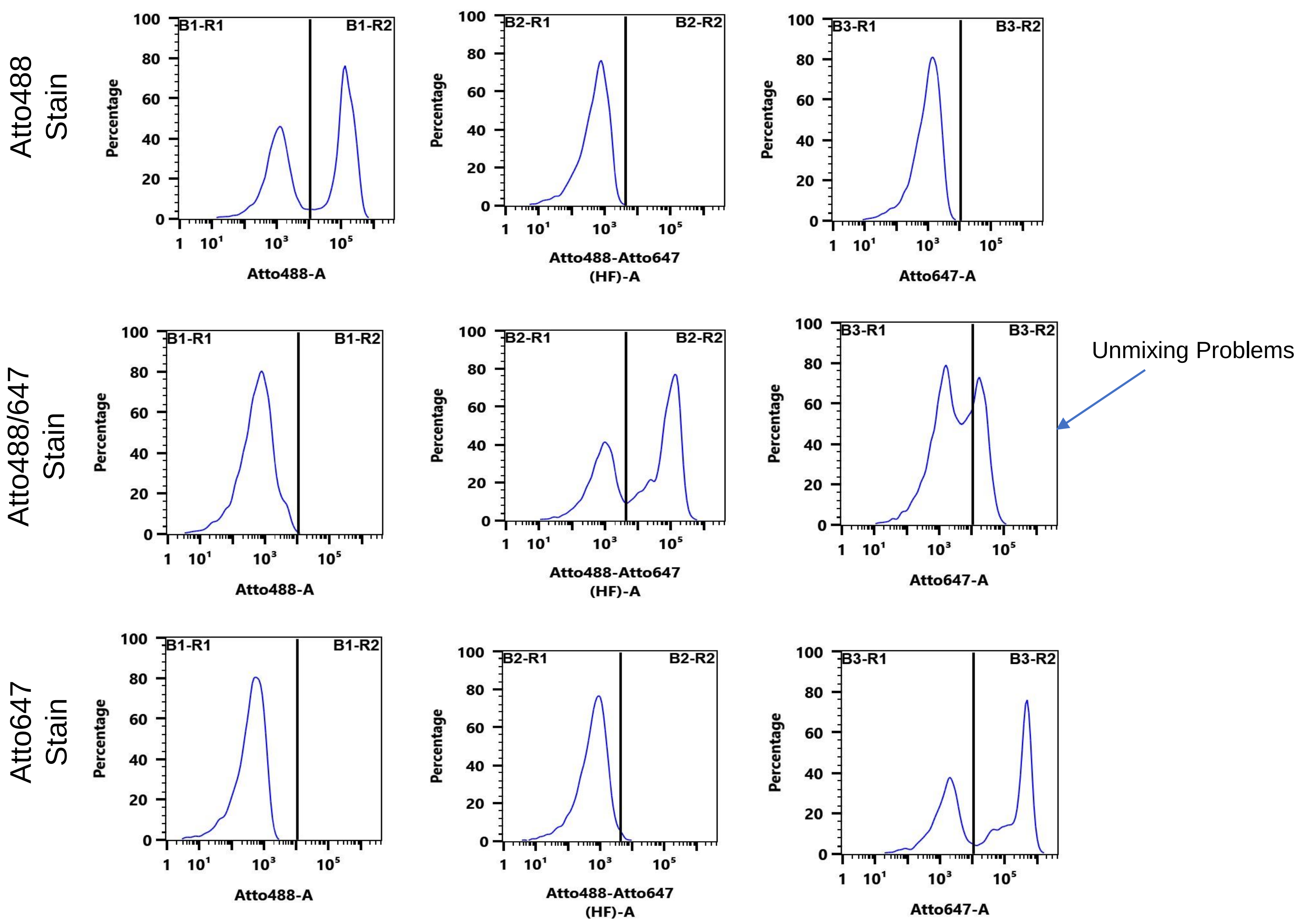

Figure S2. Unmixing Analysis of Single-Stained Cells Using Manufacturer Software. PBMCs were singly-stained as indicated, reference spectra generated, and then subjected to unmixing via the Spectroflo software. The arrow with the legend "unmixing problems" expresses the difficulties unmixing Atto488/647 single stained combination as there is a substantial positive cell population for Atto647, despite the lack of staining with that dye.

bioRxiv preprint doi: https://doi.org/10.1101/2023.07.06.547965; this version posted May 30, 2024. The copyright holder for this preprint (which was not certified by peer review) is the author/funder, who has granted bioRxiv a license to display the preprint in perpetuity. It is made available under aCC-BY 4.0 International license.

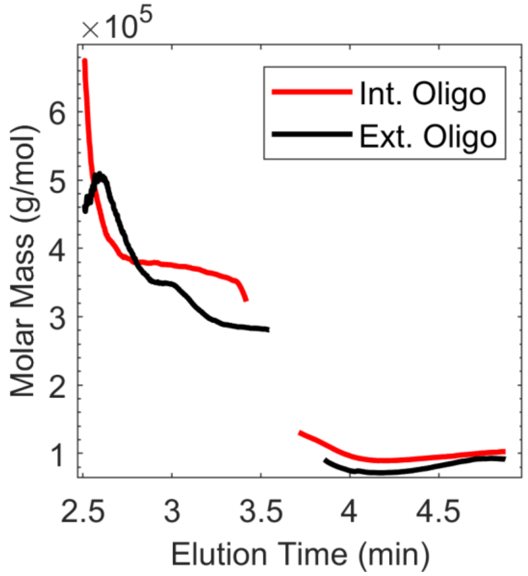

Figure S3. SEC-MALS Data for Labeled Antibody Purification. The molar mass versus elution time measured by SEC-MALS of Int. Oligo labeled antibody (red) and Ext. Oligo labeled antibody (black) solutions. Eluent from 2.5-3.5 minutes was collected for further analysis.
