# Supplementary material for: Increasing Signal Intensity of Fluorescent Oligo-Labeled Antibodies
Source: bioRxiv. 2023 Jul 6:2023.07.06.547965. Preprint. [Version 1] doi: 10.1101/2023.07.06.547965 (PMC10350089; doi:10.1101/2023.07.06.547965)
Supplement: Supplementary file 1 [file NIHPP2023.07.06.547965v1-supplement-1.pdf]

bioRxiv preprint doi: https://doi.org/10.1101/2023.07.06.547965; this version posted July 6, 2023. The copyright holder for this preprint (which was not certified by peer review) is the author/funder, who has granted bioRxiv a license to display the preprint in perpetuity. It is made available under aCC-BY 4.0 International license.

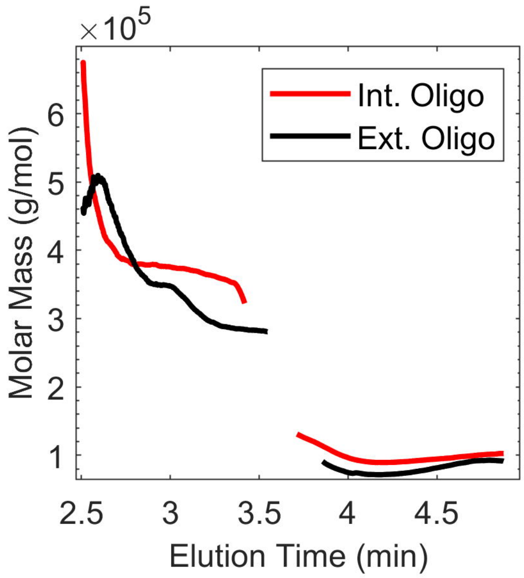

Figure S1. SEC-MALS Data for Labeled Antibody Purification. The molar mass versus elution time measured by SEC-MALS of Int. Oligo labeled antibody (red) and Ext. Oligo labeled antibody (black) solutions. Eluent from 2.5-3.5 minutes was collected for further analysis.
